# Supplementary material for: AI-Aristotle: A physics-informed framework for systems biology gray-box identification
Source: PLoS Comput Biol. 2024 Mar 12;20(3):e1011916. doi: 10.1371/journal.pcbi.1011916 (PMC10931529; doi:10.1371/journal.pcbi.1011916)
Supplement: S1 Text — X-TFC hyperparameters setup for Pharmacokinetics parameter discovery improvement. Ablation study of X-TFC for Ultradian Endocrine model. Ablation study of PINNs for Ultradian Endocrine model. (PDF) [file pcbi.1011916.s001.pdf]

Supporting Information for:

AI-Aristotle: A Physics-Informed framework for Systems Biology Gray-Box Identification

Nazanin Ahmadi Daryakenari[1◕], Mario De Florio[2◕], Khemraj Shukla[2], George Em Karniadakis[2,*]

1 Center for Biomedical Engineering, School of Engineering, Brown University, Providence, RI, USA
2 Division of Applied Mathematics, Brown University, Providence, RI, USA

◕These authors contributed equally to this work.
* george_karniadakis@brown.edu

1 X-TFC hyperparameters setup for Pharmacokinetics parameter discovery improvement

X-TFC framework shows high sensitivity to the selected hyperparameters and data, which results in very different orders of error depending on the user's selection. In this Appendix in Table A, we report improved performance for the Drug Absorption parameter discovery compared to those reported in Table 2 in the main text. Table B reports the hyperparameters used for this study, which give the best performance for parameter discovery in terms of relative error and computational time. In particular, in the case of sparse data (10 data points), we decompose the time domain in two subintervals, and a parameter estimation is performed for each of them. The resulting parameter is considered to be the average value of the two estimated parameters. For the other cases (higher resolution data), X-TFC seems to perform better for the first 25 hours of the drug absorption process, thus before the concentrations in the different compartments stabilize, reaching a steady state. We decompose the problem domain into smaller sub-domains and consider only the first one for the parameter estimation, allowing the relative errors to be reduced by several orders of magnitude.

X-TFC				
**# data points**	**relative error (%)** $\mathbf{k_g}$	$\mathbf{k_b}$	**# of iter.**	**comp. time [s]**
10	0.20	4.63	6,3	0.01
20	0.22	0.01	5	0.003
50	0.008	0.005	5	0.003
100	5.93e-06	6.99e-06	4	0.003

Table A. Pharmacokinetics model: improved performance of X-TFC for parameter discovery.

	Parameter discovery			
# data points	N	L	t_{step}	sub-domains
10	11	100	25	all
20	11	11	25	first
50	10	10	10	first
100	11	11	5	first

Table B. Pharmacokinetics model: X-TFC hyperparameters setup to reduce errors for parameter discovery and unknown term discovery.

2 Ablation study of X-TFC for Ultradian Endocrine model

In this ablation study, we evaluate the X-TFC performance for the unknown term discovery test case for the glucose-insulin model, for different distribution of input weights and bias of the neural network. The results reported in Table C are obtained using a dataset of 1800 points, time-steps of length 5 seconds, $N = 6$ collocation point per time-step, and 50 neurons, with $tanh$ as activation function. The random distributions and their probability density functions (PDF), in the range $[a, b] = [-1, 1]$, selected for this ablation study are the following:

- **Uniform distribution (unifrnd).** PDF:

$$f(x|a,b) = \begin{cases} \frac{1}{b-a} & \text{if } a \leq x \leq b \\ 0 & \text{otherwise} \end{cases} \tag{1}$$

- **Beta distribution (betarnd).** PDF:

$$f(x|\alpha,\beta) = \frac{x^{\alpha-1}(1-x)^{\beta-1}}{B(\alpha,\beta)} \tag{2}$$

where $B(\alpha, \beta)$ is the beta function defined as:

$$B(\alpha,\beta) = \int_0^1 t^{\alpha-1}(1-t)^{\beta-1}dt$$

with α and β both equal to 2.

- **Gaussian distribution (normrnd).** PDF:

$$f(x|\mu,\sigma) = \frac{1}{\sigma\sqrt{2\pi}} \exp\left(-\frac{(x-\mu)^2}{2\sigma^2}\right) \tag{3}$$

with $\mu = 0$ and $\sigma = \frac{b-a}{5}$.

- **Exponential distribution (exprnd).** PDF:

$$f(x|\lambda) = \lambda \exp(-\lambda x) \tag{4}$$

with $\lambda = \frac{b-a}{5}$.

From Table C, one can see that the variation in X-TFC performance while varying the distribution of the random weights and bias is minimal. However, we can notice that the uniform distribution is the one that gives the optimal results in terms of error. This

X-TFC						
**weights and bias**	$f(t)$			$g(t)$		
**distribution**	**MAE**	**RMSE**	**RE**	**MAE**	**RMSE**	**RE**
unifrnd	1.34e-04	5.60e-04	3.38e-05	1.37e-03	3.95e-03	1.54e-03
betarnd	1.41e-04	6.28e-04	3.78e-05	2.79e-03	7.91e-03	3.09e-03
normrnd	1.30e-04	5.54e-04	3.34e-05	3.82e-03	1.34e-02	5.22e-03
exprnd	4.38e-04	2.00e-03	1.21e-04	5.56e-03	1.52e-02	5.92e-03

Table C. Study on X-TFC performance for the unknown term discovery of glucose-insulin model, for different random distributions in input weights and bias of the neural network. The comparison is made in terms of MAE, $RMSE$, and RE, for both functions $f(t)$ and $g(t)$.

is the reason why it has been selected as the random distribution for our simulations in this paper.

Another interesting evaluation of the X-TFC performance for the unknown term discovery test case for the glucose-insulin model can be made for different activation functions of the neural network. Since we have ascertained that the uniform distribution is our optimal distribution, it is the one we use for this study. The results reported in Table D are obtained using a dataset of 1800 points, time-steps of length 5 seconds, $N = 6$ collocation point per time-step, and 50 neurons. The activation functions used for this test are:

- **Hyperbolic tangent (tanh)**

$$\tanh(x) = \frac{e^x - e^{-x}}{e^x + e^{-x}} \tag{5}$$

- **Logistic (sigmoid)**

$$\text{sigmoid}(x) = \frac{1}{1 + e^{-x}} \tag{6}$$

- **Sine activation (sine)**

$$\text{sine}(x) = \sin(x) \tag{7}$$

- **Inverse tangent (arctan)**

$$\arctan(x) = \tan^{-1}(x) \tag{8}$$

- **Softplus**

$$\text{softplus}(x) = \ln(1 + e^x) \tag{9}$$

- **Bent Identity**

$$\text{bentidentity}(x) = \frac{\sqrt{x^2+1} - 1}{2} + x \tag{10}$$

- **Inverse hyperbolic sine (asinh)**

$$\text{asinh}(x) = \ln(x + \sqrt{x^2+1}) \tag{11}$$

- **Softsign**

$$\text{softsign}(x) = \frac{x}{1 + |x|} \tag{12}$$

Also for this ablation study, from Table D we can see that all these activation functions (for the same set of hyperparameteres), allow to very similar performance in terms of error. This proves the robustness of the X-TFC method for both random distributions and activation functions. The choice of selecting *tanh* as activation function is given by the fact that it returns the best performance for the inversion of the function $g(t)$, which is the most challenging among the two functions to retrieve.

**X-TFC**						
**activation function**	$f(t)$ **MAE**	**RMSE**	**RE (%)**	$g(t)$ **MAE**	**RMSE**	**RE (%)**
tanh	1.34e-04	5.60e-04	3.38e-03	1.37e-03	3.95e-03	1.54e-01
sigmoid	3.18e-05	1.21e-04	7.29e-04	5.20e-03	1.76e-02	6.85e-01
sine	2.50e-05	8.63e-05	5.20e-04	1.91e-02	4.67e-02	1.82
arctan	1.61e-04	6.39e-04	3.85e-03	3.70e-03	1.23e-02	4.79e-01
softplus	2.54e-05	8.77e-05	5.29e-04	4.81e-03	1.64e-02	6.41e-01
bent identity	7.74e-05	3.72e-04	2.24e-03	3.84e-03	1.30e-02	5.09e-01
asinh	1.17e-04	4.86e-04	2.93e-03	3.08e-03	9.79e-03	3.82e-01
softsign	3.14e-05	1.22e-04	7.35e-04	4.15e-03	1.43e-02	5.57e-01

Table D. Study on X-TFC performance for the unknown term discovery of glucose-insulin model, for different activation functions in the neural network. The comparison is made in terms of MAE, $RMSE$, and RE, for both functions $f(t)$ and $g(t)$.

3 Ablation study of PINNs for Ultradian Endocrine model

In this comprehensive ablation study of PINNs, we present various scenarios, each characterized by distinct configurations. These configurations differ in terms of the number of neural networks employed, variations in neural network architecture, adjustments to activation functions, and experiments with varying numbers of data points.

Our study is categorized into three primary groups, each based on the number of distinct neural networks:

1. **Single Neural Network with Eight Outputs :** In this setup, a single neural network with one input and eight outputs is employed. Six of these outputs represent distinct state variables, while the remaining two outputs represent functions $f(t)$ and $g(t)$.

2. **Two Separated Neural Networks:** In this configuration, two separate neural networks are utilized—one for approximating the six state variables and another for $f(t)$ and $g(t)$.

3. **Three Separated Neural Networks:** This setup involves three distinct neural networks—one for the six state variables, one for $f(t)$, and one for $g(t)$.

The outcomes of our experimentation indicated that the first group yielded suboptimal results. The dynamics of the two unknown functions slightly deviated from the primary neural network, leading to limited improvement even with an increased number of iterations. The third group also proved to be ineffective due to extended computational times and slow learning rates, particularly for $g(t)$.

As a result, the second architecture, which employs two separate neural networks for the state variables and $f(t)$ and $g(t)$, demonstrated the most promising results. Consequently, we directed our focused investigation toward this architecture. We made adjustments to the number of collocation points, the number of data points, and the architecture of the second neural network representing $f(t)$ and $g(t)$. Furthermore, we explored different activation functions, including Rowdy (a layer-wise adaptive activation function [1]), Swish, and Tanh. The performance of PINNs in simulating a 30-hour time span is meticulously assessed and presented in Table E.

It is important to highlight that removing either the output scaling layer or the input feature layer has distinct impacts on the model's behavior. The elimination of the output scaling layer is primarily associated with convergence issues and the risk of getting stuck in local minima. On the other hand, the absence of the feature layer, even when the model is run for the same number of iterations, results in slower learning and a reduction in accuracy.

		**PINNs**			
**Act.**	**Arch.**	**# of Data**	**# of iter.**	N_c	**MAE**
Swish	32, 4	360, 360	1e06	1800	1.99e-02, 5.49e-02
Swish	32, 4	900, 360	1e06	1800	1.98e-02, 3.31e-02
Swish	20, 6	360, 360	1e06	2400	3.20e-02, 6.47e-02
Swish	32, 4	360, 360	1e06	2400	1.87e-02, 5.31e-02
Tanh	32, 4	360, 360	1e06	2400	1.06e-01, 1.48e-01
Rowdy	32, 4	360, 360	1e06	2400	1.58e-02, 7.90e-02

Table E. Comparison of PINNs with different designs. The first and second numbers in the 'Arch.' column correspond to the width and depth of the second neural network, respectively. The first and second numbers in the '# of data' column correspond to the number of data points for G and I_p, respectively. 'N_c' corresponds to Number of collocation points. In the 'MAE' column, the values correspond to the computed MAE for $f(t)$ and $g(t)$, respectively.

Performance evaluation is based on the Mean Absolute Error (MAE), calculated as described previously. The PINNs were trained with diverse configurations, encompassing different activation functions, network architectures, the number of data points, and the number of collocation points. The impact of these various configurations on the accuracy of the PINNs can be summarized as follows:

- The use of the Swish activation function yielded relatively low MAE, rendering it a favorable choice for precise simulations.
- Utilizing an increased number of data points for glucose led to further reductions in MAE for both $f(t)$ and $g(t)$.
- However, augmenting the complexity of the architecture (as observed in the 6-layer Swish configuration) did not necessarily result in improved performance, indicating a trade-off between complexity and accuracy.
- The Tanh activation function exhibited significantly higher MAE compared to Swish, indicating its limited suitability for this simulation.
- The Rowdy activation function demonstrated competitive performance with relatively low MAE, making it a viable option. However, it is essential to note that the Rowdy method entails higher computational costs.

These findings underscore the critical role of activation functions and architecture in achieving accurate simulations. The Swish activation function, in conjunction with an

appropriate architecture, emerged as the most promising configuration for this simulation.

Consequently, we leveraged the outcomes from the second row in Table E as inputs for the symbolic regression step. The computational time associated with this choice amounted to 4085.87 seconds.

References

1. Jagtap AD, Karniadakis GE. Extended Physics-informed Neural Networks (XPINNs): A Generalized Space-Time Domain Decomposition based Deep Learning Framework for Nonlinear Partial Differential Equations. In: AAAI spring symposium: MLPS. vol.~10; 2021.
